# Calcific Aortic Valve Disease: Mechanism and Future Therapeutic Strategies

**DOI:** 10.3390/cells15060542

**Published:** 2026-03-18

**Authors:** Giwon Hwang, Soyoung Jo, Hyeshin Kwon, Minjeong Kwon, Ilwhea Ku, Jae-kwan Song, Yong Hwa Jo

**Affiliations:** 1 Research and Development, REDNVIA Co., Ltd., Seoul 06675, Republic of Korea; 2 Division of Cardiology, Asan Medical Center, University of Ulsan College of Medicine, Seoul 05505, Republic of Korea

**Keywords:** cardiovascular diseases, calcific aortic valve disease, CAVD

## Abstract

**Highlights:**

**What are the main findings?**

- Calcific aortic valve disease is an actively regulated process driven by endothelial dysfunction, inflammation, extracellular matrix remodeling, and osteogenic reprogramming of valvular interstitial cells.
- Key molecular pathways and enzymes, including Notch, Wnt/β-catenin, BMP2, TGF-β, TNAP, and DPP-4, play central roles in promoting valvular calcification and disease progression.

**What are the implications of the main findings?**

- Understanding the molecular mechanisms of CAVD provides potential therapeutic targets for pharmacological intervention beyond valve replacement.
- Emerging strategies such as enzyme inhibition, RNA-based therapeutics, and targeted drug delivery may offer promising approaches to delay or prevent disease progression.

**Abstract:**

Calcific aortic valve disease (CAVD) is an active pathological process driven by complex cellular and molecular mechanisms rather than passive aging. The disease is characterized by endothelial dysfunction, lipid infiltration, inflammation, extracellular matrix remodeling, and osteogenic differentiation of valvular interstitial cells, ultimately leading to hydroxyapatite deposition and progressive valve calcification. Key signaling pathways, including Notch, Wnt/β-catenin, BMP2, and TGF-β, play critical roles in osteogenic reprogramming, while inflammatory cytokines such as IL-6, IL-1β, and TNF-α contribute to a pro-calcific microenvironment. To summarize current knowledge on CAVD pathophysiology and emerging therapeutic strategies, relevant preclinical studies were identified through searches of PubMed, and clinical trials were identified through ClinicalTrials.gov. Evidence indicates that extracellular matrix remodeling, fibrosis, and dysregulated phosphate metabolism, particularly involving TNAP and DPP-4, further accelerate disease progression. Despite advances in understanding disease mechanisms, effective pharmacological therapies remain limited, with the current treatment largely restricted to valve replacement. Emerging therapeutic approaches targeting molecular pathways, including enzyme inhibition, RNA-based therapeutics, and advanced drug delivery systems, may offer promising strategies for disease modification. A deeper understanding of CAVD pathophysiology may facilitate the development of targeted therapies to delay or prevent disease progression.

## 1. Introduction

Calcific Aortic Valve Disease (CAVD) is the most common heart valve disorder, characterized by the progressive calcification of the aortic valve, leading to aortic stenosis and various cardiovascular complications. Over the past three decades, the global burden of CAVD has grown markedly, with total cases rising from about 5 million to more than 13 million and the age-standardized prevalence rate increasing from 130.82 to 158.35 per 100,000 population [1]. It is increasingly recognized as an actively regulated pathological process rather than a passive degenerative condition associated with aging [2]. The epidemiology of CAVD highlights its rising prevalence, particularly among the aging population, with estimates suggesting that over 10% of individuals aged 75 and older are affected [3]. This significant burden not only impacts patient quality of life but also places a substantial strain on healthcare resources.

The pathophysiology of CAVD is complex, involving a multifactorial interplay of cellular and molecular mechanisms. Initial stages of the disease are marked by endothelial injury and lipid infiltration, which trigger a cascade of inflammatory responses and extracellular matrix (ECM) remodeling. As the disease progresses, valve interstitial cells (VICs) undergo osteogenic reprogramming, leading to the formation of calcific nodules and ultimately resulting in severe restriction of leaflet mobility [4]. Understanding these underlying mechanisms is crucial for developing effective therapeutic interventions.

Current standard treatments for CAVD include surgical aortic valve replacement (SAVR) and transcatheter aortic valve replacement (TAVR). While these procedures can significantly improve hemodynamics and patient outcomes, they do not address the underlying disease process. Patients, particularly the elderly or frail, face substantial risks associated with these interventions, and the durability of prosthetic valves is a persistent concern, often necessitating reintervention [5,6]. The limitation of these invasive treatment options has increased the need for the development of therapeutic drugs.

Given the limitations of current therapeutic approaches, there is an urgent need for new strategies to target the underlying mechanisms of CAVD. Insights into the active nature of this disease, including its association with chronic inflammation, fibrosis, and active calcification, provide a strong rationale for the development of targeted pharmacological interventions. Investigational therapies are increasingly focusing on modulating specific molecular pathways implicated in the disease, such as the IL-6 signaling, Notch/Wnt/BMP2 pathways, TNF-α/TGF-β signaling and the osteogenic differentiation of VICs [7]. Additionally, drug repurposing strategies, alongside novel delivery systems and RNA therapeutics, present promising avenues for addressing this condition.

The purpose of this review is to provide a comprehensive overview of CAVD, encompassing its epidemiology, pathophysiology, clinical significance, and current treatment strategies. We will critically evaluate the limitations of existing therapies and explore the necessity for innovative treatment approaches. Furthermore, we will discuss ongoing clinical trials and investigational therapeutic strategies that hold promise for the future management of CAVD. By synthesizing the latest research findings and clinical data, this review aims to inform clinicians and researchers about the evolving landscape of CAVD management and to highlight opportunities for advancing therapeutic interventions that could significantly improve patient outcomes.

## 2. Pathophysiology of CAVD: A Brief Overview

CAVD is no longer regarded as a passive degenerative process of aging, but rather as an actively regulated pathology governed by cellular and molecular mechanisms. Disease progression unfolds through a multistep pathophysiological sequence, beginning with early endothelial injury and lipid infiltration, followed by inflammation and ECM remodeling, and progressing to late-stage events characterized by VICs’ osteogenic differentiation, fibrosis, and the transition from micro- to macro-calcification [8]. These mechanisms operate in a mechanically active environment, with significant interplay between calcification and fibrosis contributing to progressive valve stiffening and malfunctioning [9].

### 2.1. Cellular Dysfunction and Inflammation

Aortic valve leaflets are exposed to continuous motion and substantial mechanical stress, and in calcific aortic valve disease, the fundamental pathogenic mechanism is initiated when valvular interstitial cells transdifferentiate into osteoblast-like cells. VICs adjacent to the injured VECs were directly exposed to mechanical stress, including shear stress [9]. Immune cells infiltrated the aortic valve tissue and triggered an inflammatory response [10]. Following this inflammation, the aortic valve leaflets developed swelling due to inflammation and underwent ECM remodeling [11]. Although these processes initiated the development of valvular disease, there were still no clinical symptoms [12].

### 2.2. Fibrosis and Aortic Valve Stiffness

Following the initial inflammatory phase, aortic valve leaflets undergo progressive fibrosis characterized by myofibroblastic activation of VICs. These activated VICs deposit excessive collagens (primary types I and III) and proteoglycans, disrupting ECM structure. This dysregulated ECM turnover, driven by upregulated matrix metalloproteinases (MMPs) and tissue inhibitors of metalloproteinases (TIMPs), leads to net matrix accumulation and early leaflet thickening without immediate hemodynamic compromise [13,14].

Fibrotic remodeling directly contributes to aortic valve stiffness through collagen crosslinking mediated by lysyl oxidase and accumulation of glycosaminoglycans, which reduce tissue compliance and impair systolic excursion. Disrupted mechano-transduction from injured VECs perpetuates VIC activation in a feed-forward loop, amplifying stiffness that elevates transvalvular gradients over time [15]. TGF-β signaling pathways amplify this process by sustaining myofibroblast differentiation and inhibiting ECM degradation [16]. This fibroelastic phase often precedes manifest calcification, representing a critical window for therapeutic intervention targeting matrix homeostasis.

### 2.3. Calcification: Terminal Phase of CAVD

Progressing from the fibroblastic phase, calcification constitutes the terminal pathophysiological stage of CAVD, marked by pathological osteogenic differentiation of VICs into osteoblast-like phenotypes. These activated VICs express bone-related proteins such as alkaline phosphatase, osteopontin, osteocalcin, and BMP-2/4, driving hydroxyapatite deposition and formation of calcific nodules within the valve matrix [17]. This biomineralization process progressively stiffens the leaflets, transforming compliant tissue into a bone-like structure that severely restricts systolic opening [18].

Extracellular vesicles from apoptotic VICs and macrophages promote the characteristic progression from micro- to macro-calcification on the fibrotic scaffold. Mechanical stiffness from prior fibrosis further accelerates this osteogenic progression in a feed-forward loop, culminating in critical aortic stenosis, elevated transvalvular gradients, and heart failure symptoms that mandate surgical replacement [14,18].

## 3. Therapeutic Implication Based on Pathophysiological Mechanisms

### 3.1. Physiological Molecular Pathways

#### 3.1.1. Endothelial Dysfunction and Lipid Infiltration

**Figure 1:**
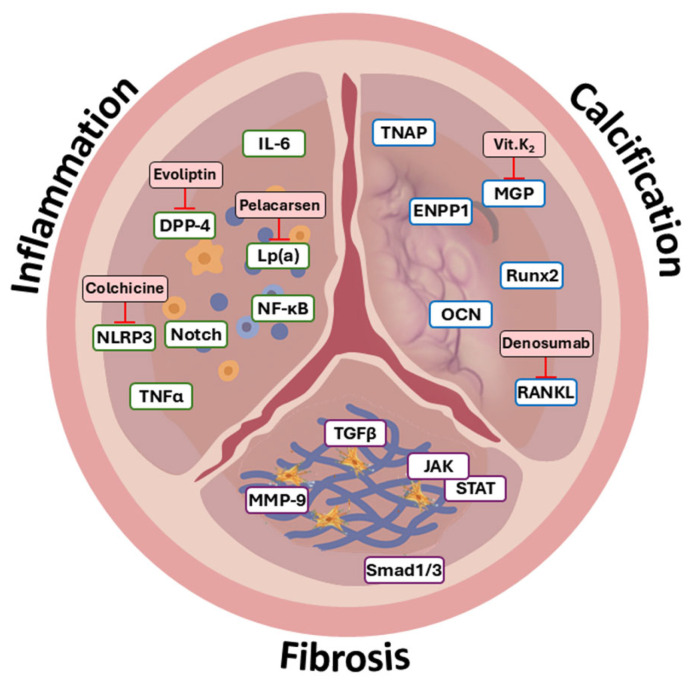
Pathological features and molecular pathways in aortic valve disease. Schematic illustration of the aortic valve showing inflammation, fibrosis, and calcification. White boxes indicate key genes and signaling pathways involved in each pathological stage, while pink boxes represent inhibitors or therapeutic candidates under clinical or preclinical investigation.

The aortic valve is continuously exposed to hemodynamic stress, with the fibrosa side being particularly susceptible to oscillatory shear stress and disturbed flow, rendering it prone to endothelial injury [14]. Under physiological conditions, valve endothelial cells (VECs) secrete nitric oxide (NO) and prostacyclin to maintain an anti-inflammatory, anti-thrombotic, and anti-calcific environment. However, alterations in shear stress and the accumulation of reactive oxygen species (ROS) lead to endothelial dysfunction, resulting in the loss of these protective functions [19,20]. Through the damaged endothelium, low-density lipoprotein (LDL), oxidized phospholipids (oxPL), and lipoprotein(a) [Lp(a)] infiltrate into the subendothelial space. Oxidized low-density lipoprotein (oxLDL) and Lp(a) bind strongly to proteoglycans, leading to their retention around VICs. Beyond structural lipid deposition, these lipid species actively reshape cellular metabolism within VICs and macrophages, promoting glycolytic shifts and inflammatory energy demands that further accelerate calcific signaling. This process is accompanied by upregulation of VCAM-1 and ICAM-1, which promote monocyte recruitment and macrophage infiltration [20,21]. Infiltrated macrophages differentiate into foam cells and release pro-inflammatory cytokines, IL-6, TNF-α, as well as matrix metalloproteinases (MMPs), thereby driving ECM remodeling and further endothelial injury [22]. These early lesions share pathological similarities with atherosclerosis [23]. However, unlike blood vessels, the aortic valve lacks vasa vasorum, is subjected to persistent mechanical strain, and harbors a collagen-rich microenvironment, resulting in distinct disease progression. Specifically, while a lipid-rich necrotic core is frequently observed in atherosclerosis, CAVD is characterized by osteogenic differentiation and the formation of calcific nodules as dominant pathological features [23,24] (Figure 1).

#### 3.1.2. Inflammation, ECM Remodeling, and Fibrosis

Lipid infiltration, such as oxLDL and Lp(a), rapidly triggers the recruitment of macrophages (predominantly M1 phenotype) and T helper lymphocytes (T_H_1/T_H_17 subsets) into the aortic valve. These immune cells secrete cytokines, including IL-1β (interleukin-1β), IL-6, TNF-α (tumor necrosis factor-α), IFN-γ (interferon-γ), and TGF-β1 (tumor growth factor-β1), thereby promoting the myofibroblast-like activation of VICs and their osteogenic reprogramming characterized by the upregulation of RUNX2 (runt-related transcription factor 2), ALPL (alkaline phosphatase, biomineralization-associated), and BMP2 (bone morphogenic protein 2). These inflammatory cytokines cascade converge on the NF-κB, JAK-STAT, and TGF-β–SMAD signaling, driving gene programs involved in ECM remodeling and amplifying both fibrosis and calcification [25].

In addition, Toll-like receptors, TLR2 and TLR4, expressed on VICs, VECs, and infiltrating immune cells, recognize danger-associated molecular patterns (DAMPs)—including fragmented ECM components, elastokines, and oxLDL-leading to NF-κB activation and induction of pro-inflammatory cytokines and MMPs [26]. Activation of the NLRP3 (NOD-, LRR- and pyrin domain-containing protein 3) inflammasome within valvular lesions promotes caspase-1-dependent maturation of IL-1β and IL-18, thereby promoting the osteogenic differentiation of VICs. Pharmacological inhibition of NLRP3 attenuates calcification in preclinical models [27]. Among ECM-derived DAMPs, biglycan—a small leucine-rich proteoglycan elevated in calcified valves—binds TLR2/4, inducing BMP2 and TGF-β1 signaling that converges on Smad1/3 phosphorylation. This induces osteogenic markers including ALP, Runx2, and osteopontin, alongside calcium deposition—effects abolished by dual inhibition of BMP2 and TGF-β1 [28]. These findings implicate ECM-derived matricellular proteins as active modulators linking innate immune responses to osteogenic differentiation in CAVD.

Fibrotic remodeling, characterized by increased collagen deposition and myofibroblast activation, enhances ECM stiffness and reconfigures the valvular microenvironment. The alters matrix promotes VICs’ sensitivity to pro-calcific stimuli, accelerating expression of osteogenic genes such as RUNX2, BMP2, and ALPL. Rather than passive consequence, fibrosis operates as a dynamic facilitator of calcification, establishing a mechanobiological feedback loop that sustains and amplifies disease progression [9].

#### 3.1.3. Osteogenic Differentiation of VICs and Calcification

In the terminal stage of calcification, VICs undergo trans-differentiation into osteoblast-like cells, driving hydroxyapatite deposition and the formation of calcific nodules within the valve leaflet. These lesions initially manifest as microcalcifications but progressively expand into nodular macro-calcifications over time, mechanically restricting leaflet mobility [29]. Macro-calcifications contribute to increased hemodynamic stress, which is increasing mechanical stress and thereby promoting sustained VECs dysfunction and reactivation of VICs in a pathological feedback loop [30]. A pivotal molecular catalyst in this process is tissue-nonspecific alkaline phosphatase (TNAP, genetic name; ALPL). TNAP hydrolyzes pyrophosphate (PPi) into inorganic phosphate (Pi), thereby accelerating hydroxyapatite nucleation. As VICs undergo osteogenic reprogramming, TNAP expression and activity are upregulated, shifting the extracellular Pi/PPi ratio toward a calcification-prone state, and then hydroxyapatite crystal growth acts as a catalyst for the transition from micro-calcification to nodular macro-calcification [31]. Thus, VIC-driven calcification should be understood not as a passive mineral deposition but as a positive feed-forward loop encompassing VICs’ osteogenic differentiation, TNAP activation, Pi accumulation, hydroxyapatite crystal growth, mechanical stress, and recurrent VIC/VEC injury [31,32,33].

#### 3.1.4. Osteogenic Differentiation of VICs

DPP-4 axis. Several studies, including those from our group, have demonstrated that dipeptidyl peptidase-4 (DPP-4) serves as an important upstream regulator of VICs’ osteogenic differentiation. Endothelial nitric oxide (eNOS) deficiency activates the NF-κB pathway, leading to increased expression of DPP-4 in VICs. This, in turn, suppresses insulin-like growth factor-1 (IGF-1) signaling and promotes a Runx2-centered osteogenic gene program. Indeed, as reported in *Circulation* [34], pharmacological inhibition of DPP-4 with sitagliptin effectively blocked osteoblastic trans-differentiation of VICs in vitro and significantly reduced aortic valve calcification in both eNOS^−/−^ mice and hypercholesterolemic rabbits fed vitamin D2. These findings indicate that DPP-4 overexpression contributes to the development and progression of CAVD, highlighting its potential as a therapeutic target [34,35].

TNAP–Pi axis. Another critical driver of the osteogenic differentiation of VICs is TNAP. TNAP promotes calcification by hydrolyzing inorganic PPi, a potent endogenous inhibitor of mineralization, thereby reducing its protective effect [36]. At the same time, TNAP hydrolyzes β-glycerophosphate (β-GP) to generate Pi [37]. This dual action shifts the extracellular Pi/PPi ratio toward a calcification-prone environment, thereby amplifying the expression of osteogenic markers such as OCN (osteocalcin), ALPL, and RUNX2 in VICs [38,39]. The accumulated Pi enters VICs via the sodium-dependent phosphate transporter PiT-1, which activates Akt-1 signaling and further drives osteogenic differentiation. Consistent with this, human CAVD valve tissues exhibit elevated PiT-1 expression, and silencing Akt-1 with siRNA markedly reduces Pi-induced mineralization of VICs [40].

### 3.2. CAVD Pathological Key Molecules

#### 3.2.1. IL-6

IL-6 functions as a key regulator of inflammatory signaling pathways in CAVD [41]. IL-6 affects both VICs and immune cells in the calcification process in CAVD. First, it promotes the expression of osteogenic transcription factors, RUNX2 and SP7 (osterix), by activating the gp130/STAT3 pathway, thereby accelerating the trans-differentiation of VICs into an osteoblast-like phenotype. STAT3 directly induces the expression of ALPL, COL1A1, and BMP2, providing the molecular foundation for calcific nodule formation [42]. Furthermore, IL-6 modulates the OPG/RANKL axis, disrupting the balance of osteoclast-like activity. Under physiological conditions, OPG antagonizes RANKL to regulate bone resorption; however, within CAVD lesions, IL-6 downregulates OPG while upregulating RANKL, thereby triggering osteoclast-like differentiation in VICs and infiltrating macrophages. This imbalance promotes the growth of calcified nodules [43,44]. Second, at the immune cell level, activated macrophages and T_H_17 cells secrete large amounts of IL-6, which in turn reinforces osteogenic reprogramming of VICs through a positive feedback loop. Notably, the IL-6/IL-17 produced by T_H_17 cells has been shown to sustain a chronic inflammation that persistently stimulates VICs’ calcification [45]. Thus, IL-6 acts not only as a pro-inflammatory cytokine but as a central hub that interconnects inflammation, osteogenic reprogramming, and calcification in the pathogenesis of CAVD.

#### 3.2.2. Notch, Wnt and BMP2 Signaling

The Notch/Wnt/BMP2 signal, well-established as the canonical pathway of osteogenesis, similarly mediated the osteoblast-like reprogramming of VICs in CAVD. Notch1 confers anti-calcific protection on healthy VECs and VICs by inhibiting the transcription of RUNX2, an osteogenic regulator. Conversely, mutations or reductions in Notch1 relieve this inhibitory control, resulting in RUNX2 upregulation and promoting pathological calcification [46]. Notch haploinsufficiency has been identified as a strong genetic basis for familial early-onset CAVD [47]. CAVD is related to prototypical extracellular signaling, such as Wnt/β-catenin and BMP/Smad pathways. Engagement of Wnt ligands with Frizzled receptors and the LRP5/6 co-receptors prevents β-catenin degradation, allowing β-catenin to translocate into the nucleus where it binds to lymphoid enhancement factor/T-cell factor (LEF/TCF) transcription factors and activates osteogenic genes, including RUNX2, ALPL, and OCN. This process is accelerated by ECM stiffness and mechanical stress, which act synergistically to drive VICs osteogenic differentiation [48,49]. BMP2, osteoinductive cytokines, promotes RUNX2 and TNAP expression through activation of SMAD1/5/8 signaling [50]. In addition to promoting the osteogenic transition of VICs, BMP2 also enhances the release of calcified extracellular vesicles (EVs), thereby facilitating hydroxyapatite formation. Importantly, inflammatory cytokines such as IL-1β and TNF-α, as well as oxidative stress, activate the BMP2 promoter, as a central mediator of the inflammation–calcification.

#### 3.2.3. TNF-α and TGF-β1

Among inflammatory mediators, TNF-α is recognized as one of the most potent pro-calcific cytokines in CAVD. TNF-α activates NF-κB and JNK signaling cascades, thereby promoting both myofibroblast-like activation and osteogenic differentiation of VICs [51]. TNF-α stimulation induces the expression of RUNX2, a master osteogenic transcription factor, and upregulates osteogenic markers such as ALP and OSX (osterix), ultimately accelerating calcification [52]. In addition, TNF-α establishes an autocrine inflammatory loop in VICs that amplifies the secretion of IL-1β and IL-6, promoting macrophage and T-cell infiltration, perpetuating an inflammation–calcification feed-forward loop [42].

In parallel, TGF-β1 serves as a dual regulator that integrates fibrotic and osteogenic signaling. Through SMAD2/3 phosphorylation, TGF-β1 drives the myofibroblast activation of fibroblast-like VICs, leading to increased expression of ECM components, including α-SMA and collagen I [28]. Concurrently, activation of SMAD1/5/8 signaling promotes osteogenic differentiation and cooperates with BMP2-RUNX2 signaling to potentiate osteogenesis [49]. This bifurcated signaling demonstrates that fibrosis and calcification are not independent or sequentially distinct processes but represent interconnected pathological programs.

Thus, TNF-α and TGF-β1 correlate with a pathogenic process in which VICs undergo progressive phenotypic reprogramming- from fibrosis-like to myofibroblast-like and ultimately to osteoblast-like states. During the early phase, TGF-β1–SMAD2/3 signaling predominates, driving ECM remodeling of the VICs. Subsequently, activation of TNF-α and TGF-β1–SMAD1/5/8 pathways initiate RUNX2-mediated osteogenic reprogramming. This sequential transition provides a mechanistic explanation of how fibrosis and calcification are interconnected through a feed-forward loop that accelerates disease progression in CAVD.

#### 3.2.4. TNAP

TNAP is a key enzyme in normal bone formation that regulates phosphate homeostasis by hydrolyzing PPi, as a known inhibitor of calcification, thereby increasing Pi levels and promoting hydroxyapatite formation [53]. In CAVD, pathological overexpression of TNAP has been observed, and vesicle-associated TNAP released from VICs and macrophages induces matrix vesicle-mediated calcification [38,54]. TNAP acts as a downstream effector of RUNX2 and BMP2 signaling, driving the osteogenic program toward the terminal mineralization stage. Importantly, TNAP activity functions at the crossroads of fibrosis and calcification, integrating pathological signaling from both processes. Its enzymatic activity is enhanced within collagen-rich, stiff ECM environments, where it synergizes with fibroblast-to-myofibroblast transition to exacerbate ectopic calcification [55,56]. Thus, while TNAP is indispensable for physiological bone mineralization, it also serves as a pivotal pathological biomarker and potential therapeutic target in ectopic calcification, including CAVD [54].

### 3.3. The Importance of Drug Development in CAVD

Despite the growing understanding of the cellular and molecular mechanisms underlying CAVD, effective pharmacological options remain unavailable. Currently, SAVR and TAVI represent the only established therapies. While these procedures improve hemodynamics and survival, they do not address the underlying disease process. Moreover, surgical approaches are associated with substantial risks in elderly or frail patients, and prosthetic valves have limited durability, frequently requiring reintervention [6,57].

Several clinical trials have investigated statins, angiotensin-converting enzyme inhibitors, bisphosphonates, and other agents, but none have demonstrated efficacy in halting disease progression [58,59]. This lack of disease-modifying therapy highlights a critical unmet medical need. As the global population ages, the prevalence of CAVD continues to rise, with estimates suggesting that more than 10% of individuals over 75 years of age are affected [3]. The socioeconomic burden of surgical management is therefore expected to escalate, emphasizing the necessity of alternative therapeutic strategies.

## 4. Clinical Trial Updates in CAVD

### 4.1. Clinical Trials Overview

**Figure 2:**
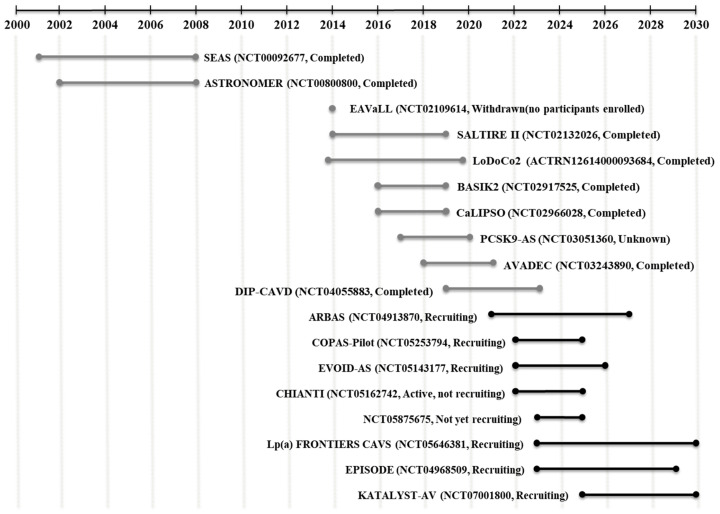
Timeline of CAVD clinical trials, 2000–2030. Grey line, completed or withdrawn trials; Black line, ongoing trials.

To date, valve replacement surgery remains the only effective therapeutic option for CAVD. Over the past decades, various therapeutic strategies, including drug repurposing and the exploration of novel molecular targets, have been evaluated in clinical trials. Although most of these studies have not demonstrated significant efficacy, an increasing number of ongoing trials highlights the growing potential for the development of effective pharmacological therapies for CAVD. Herein, we review and discuss the current landscape of clinical trials investigating medical treatments for CAVD (2000–2030, PubMed/ClinicalTrials.gov) (Table 1). Figure 2 provides an overall schematic illustration of the temporal landscape and mechanistic evolution of CAVD clinical trials, and Table 1 presents detailed information on individual studies, including their phases, indications, primary endpoints, and sponsors.

### 4.2. Past Clinical Trials

To evaluate trends in clinical trials, studies conducted between 2000 and 2030 were identified through searches of ClinicalTrials.gov. Trial information was further verified using published literature in PubMed and information available from sponsors’ websites or official releases. The search terms included CAVD, aortic valve calcification, aortic stenosis, and calcific aortic valve stenosis. Eligible studies included completed or ongoing trials assessing disease progression using imaging or hemodynamic parameters. Trials involving surgical or device-based interventions, or those not directly related to disease progression, were excluded.

Early clinical trials mainly focus on lipid-lowering therapies, such as statins (HMG-CoA inhibitors), but they failed to demonstrate the expected clinical benefits. For example, in the SEAS trial (NCT00092677), combination therapy with simvastatin and ezetimibe reduced LDL levels by approximately 61%, yet it did not decrease the incidence of major cardiovascular events or slow the progression of aortic valve stenosis [59]. Similarly, there was no significant difference in the rise in peak aortic valve gradient or the reduction in valve area by treatment with rosuvastatin 40 mg compared with placebo in the ASTRONOMER trial (NCT00800800) [60]. Taken together, these two studies consistently confirmed that statin-based lipid-lowering therapies are ineffective in slowing the progression of aortic stenosis. This recognition redirected clinical and research attention toward the pathological calcification underlying CAVD.

Even though vitamin K2 supplements are known to support blood clotting and bone health, and to prevent arterial calcification by activating matrix Gla protein (MGP), which inhibits calcium deposition in blood vessel walls, clinical trials, as BASIK2 (NCT02917525) and AVADEC (NCT03243890), were failed to demonstrate reductions in aortic valve calcium (AVC) progression, valve area decline, or velocity changes [61].

Clinicians and researchers focused on osteoporosis therapeutic drugs because some of these drugs increase the resorption or decrease in phosphate in the blood [62]. However, in the SALTIRE II trial (NCT02132026), neither denosumab nor alendronic acid produced a significant difference in CT-based aortic valve calcium (CT-AVC) scores compared with placebo over 24 months [63].

More recently, new mechanism-based approaches have been explored. The CaLIPSO trial (NCT02966028) investigated SNF472, which directly inhibits hydroxyapatite crystallization, to delay cardiovascular calcification. In end-stage renal disease (ESRD) patients with dialysis, SNF472 significantly attenuated the progression of coronary artery calcium and aortic valve calcification [64]. In the CALCIPHYX trial (NCT04195906), SNF472 failed to show statistically significant improvements in the two primary efficacy endpoints—calciphylaxis wound healing and pain—compared with placebo. Secondary endpoints, including time to complete wound healing, change in pain scores, and health-related quality of life, also did not achieve, leading to failure in demonstrating clear clinical efficacy [65]. Therefore, despite its initial ability to inhibit coronary and aortic valve calcification, SNF472 has not progressed to large-scale phase 3 studies in CAVD patients due to its failure to establish convincing clinical benefits in calciphylaxis.

### 4.3. Emerging Mechanism-Driven and Precision-Targeted Clinical Trials in CAVD

In recent years, clinical studies have increasingly focused on mechanism-based, precision-targeted therapeutic strategies, as metabolic disorders such as diabetes and hyperlipidemia have been identified as major contributors to the pathogenesis of CAVD.

NCT05875675 is investigating the potential therapeutic role of pioglitazone, an anti-diabetic thiazolidinedione that acts as a peroxisome proliferator-activated receptor gamma (PPAR-γ) agonist, in CAVD. Through activation of PPAR-γ, pioglitazone exerts anti-inflammatory and anti-fibrotic effects that may suppress VICs activation and ECM remodeling, both of which are critical steps in valvular calcification. These pharmacologic properties provide a mechanistic rationale for evaluating pioglitazone as a disease-modifying therapy for CAVD. Preclinical evidence suggests that PPAR-γ activation can inhibit TGF-β–driven profibrotic signaling and attenuate inflammatory cytokine production, supporting its repurposing potential [66,67]. The ongoing phase 2 trial aims to determine whether pioglitazone can slow disease progression in patients with mild-to-moderate aortic stenosis by targeting these pathological pathways.

The DIP-CAVD trial (NCT04055883) first introduced the potential of the DPP-4 inhibitor, evogliptin, to inhibit aortic valve calcification. Although long-term follow-up data did not show a significant difference in CT-based aortic valve calcium volume (AVCV) compared with placebo, ^18^F-NaF PET imaging revealed a significant reduction in active calcification markers, providing encouraging evidence [68]. Building on these findings, the follow-up EVOID-AS trial (NCT05143177) is now underway. This study aims to more clearly define the effect of evogliptin on AVCV by incorporating CT-based Agatston scores as the primary endpoint, thereby addressing limitations of the previous study and enabling more definitive conclusions.

Elevated Lp(a) has been consistently identified in both genetic and clinical studies as an independent risk factor for CAVD [69,70]. Increased Lp(a) levels promote valvular calcification not only through lipid deposition but also by amplifying local inflammatory signaling within valvular tissue.

Building on this pathogenic rationale, pelacarsen—an antisense oligonucleotide (ASO) that selectively targets Lp(a)—was developed to directly lower circulating Lp(a) concentrations. The ongoing phase 2 trial (NCT05646381) is enrolling patients with aortic stenosis and elevated Lp(a) levels to determine whether pelacarsen can slow the progression of valve calcification and hemodynamic deterioration compared with placebo. The study’s primary endpoints are changes in peak aortic jet velocity (assessed by echocardiography) and AVC scores quantified by computed tomography. Although its clinical efficacy remains to be demonstrated, pelacarsen represents a highly promising disease-modifying candidate—supported by robust genetic evidence—poised to become the first targeted therapeutic approach for CAVD.

The proprotein convertase subtilisin/kexin type 9 (PCSK9) inhibitor trial (NCT04968509) is a phase 3 study conducted in patients with aortic stenosis who are already receiving statin therapy. This study aims to determine whether PCSK9 inhibition can more effectively reduce LDL-C and Lp(a) levels, and whether these lipid-lowering and anti-inflammatory effects can slow disease progression. PCSK9 inhibitors not only potently lower LDL-C, but also reduce Lp(a), attenuate inflammatory responses, and suppress atherosclerotic processes—suggesting benefits beyond lipid-lowering. The trial evaluates echocardiographic hemodynamic indices (Vmax, AVA) alongside CT-based AVC scoring to assess whether these effects translate into clinically meaningful inhibition of stenosis progression. While previous statin-based trials failed to demonstrate efficacy against valvular calcification, PCSK9 inhibition is anticipated as a novel therapeutic strategy with the potential to delay disease progression by addressing both lipid and inflammatory pathways. KATALYST-AV trial (NCT07001800) is a phase 3 trial evaluating the soluble guanylate cyclase (sGC) activator, ataciguat, in adults with moderate calcific aortic valve stenosis (CAVS). The trial was designed based on results from the earlier phase 2 study of ataciguat (NCT02481258), which failed to reach statistical significance due to a small sample size and limited follow-up duration [71]. Nevertheless, the study demonstrated a trend toward reducing the progression of valve calcification by approximately 69.8% compared with placebo. On this basis, a subsequent phase 3 trial was initiated to revalidate ataciguat in the form of an indication patent (drug repurposing), as its original composition patent had already expired.

Colchicine is a well-known anti-inflammatory drug that inhibits tubulin polymerization and consequently blocks activation of the NLRP3 inflammasome, a pathway involved in chronic inflammation that promotes calcification. The CHIANTI trial (NCT05162742) is a phase 3 study evaluating colchicine as an anti-inflammatory therapy to slow the progression of aortic stenosis in patients with mild-to-moderate disease. The trial assesses changes in CT-AVC scores, as well as echocardiographic parameters including peak aortic jet velocity and effective orifice area. However, adverse events such as gastrointestinal intolerance and myotoxicity have been reported, particularly in elderly patients and those with renal impairment, which warrants caution [72]. Evidence from broader cardiovascular cohorts also supports the therapeutic potential of colchicine as an anti-inflammatory agent. In the LoDoCo2 trial (ACTRN12614000093684), low-dose colchicine (0.5 mg/day) was associated with a significant reduction in major adverse cardiovascular events [72].

The COPAS-Pilot trial (NCT05253794) is a small, randomized, placebo-controlled study designed to assess the efficacy of colchicine in patients with aortic stenosis. The trial is based on the hypothesis that inhibition of inflammatory signaling can reduce the metabolic activity underlying valvular calcification. Unlike previous trials that primarily focused on structural progression using CT-based AVC scoring, COPAS employs ^18^F-NaF positron emission tomography (PET) imaging to quantify active calcification. This approach enables detection of early therapeutic responses and assessment of pharmacodynamic effects within a short-term, six-month follow-up period. By targeting inflammation-driven mechanisms, the study represents one of the first attempts to evaluate systemic anti-inflammatory therapy as a disease-modifying strategy for CAVD. Beyond these, additional trials are exploring diverse mechanisms. The ARBAS trial (NCT04913870) is investigating angiotensin receptor blockers (ARBs) in patients with mild-to-moderate aortic stenosis, assessing whether ARB therapy can reduce the rate of aortic valve calcification progression and attenuate left ventricular remodeling.

## 5. Investigational Therapeutic Approaches in CAVD

CAVD progresses through a multistep biological cascade characterized by chronic inflammation, fibrosis, ECM remodeling, and ultimately pathological calcification. To replicate and quantitatively assess these complex pathophysiological processes at the preclinical stage, appropriate animal models capable of reproducing and analyzing these conditions are indispensable. Based on these experimental findings, several therapeutic strategies have been investigated to target the major pathogenic mechanisms of CAVD. These approaches involve diverse mechanisms.

To summarize emerging therapeutic strategies for CAVD (Table 2), relevant studies published from 2015 to the present were identified through searches of PubMed, ClinicalTrials.gov, and related published literature. The literature search focused on studies investigating CAVD pathophysiology, recently emerging therapeutic targets, and potential therapeutic approaches. The search terms included CAVD, calcific aortic valve disease, aortic valve calcification, vascular calcification, valvular interstitial cell, VIC osteogenic differentiation, inflammation, fibrosis, drug repurposing, gene therapy, and cell therapy. Eligible studies included preclinical investigations (in vitro or in vivo models) and clinical studies evaluating molecular mechanisms or therapeutic strategies targeting CAVD or valvular calcification. Studies unrelated to CAVD pathogenesis, observational studies without mechanistic or therapeutic evaluation, surgical or device-based interventions, and studies lacking experimental or therapeutic relevance were excluded.

### 5.1. Small Molecules

Various molecules associated with osteogenic signaling have been proposed as major targets in the progression of CAVD. First, RUNX2, BMP2, and Wnt, known as osteogenic markers and key components of osteogenic pathways, have remained important preclinical targets [7]. Second, pathological calcification signaling pathways involving ENPP1 and TNAP promote ectopic mineralization by increasing phosphate levels and reducing PPi [73]. Third, SNF472, a small-molecule inhibitor of hydroxyapatite crystallization, has demonstrated potent anti-calcific efficacy by preventing mineral nucleation and crystal growth in experimental models and has shown reduced progression of cardiovascular calcification in clinical studies [64]. Finally, integrated proteomic and metabolomic analyses have identified the tryptophan, kynurenine pathway and choline metabolism as key regulators of osteogenic differentiation and calcification progression [86].

### 5.2. Gene Therapy

Genetic predisposition is closely associated with CAVD development, and specific genetic variations are considered therapeutic targets. Variants in NOTCH1, ENPP1, and PALMD have been related to early-onset calcification [87,88]. Multi-omics studies have further identified LPA, RUNX2, BMP2, and Wnt pathways as critical targets [7].

Based on these results, gene editing offers a novel therapeutic approach for treating CAVD. RNA therapeutics provides a direct approach to targeting genes implicated in CAVD. Especially, the regulation of non-coding RNAs can mitigate the osteogenic differentiation of VICs. Inhibition of miR-34a disrupts the NOTCH1–RUNX2 axis and decreases VICs osteogenic differentiation, making it a potential therapeutic target supported by cellular and animal studies [74]. Conversely, lncRNA H19 is upregulated in valve tissues of CAVD patients. It promotes osteogenic differentiation by interfering with the Notch pathway via promoter hypomethylation and NOTCH1 inhibition. Therefore, inhibition of lncRNA H19 may confer anti-calcification effects [75].

CRISPR-Cas9 technology has been employed to precisely edit or suppress genes implicated in calcification, demonstrating significant anti-calcification effects in vitro and in vivo. While RNA therapies enable transient modulation, CRISPR offers the potential for permanent gene correction, providing prospects for long-term therapeutic benefits [76].

### 5.3. Cell Therapy

Cell-based therapeutic strategies in CAVD primarily focus on mesenchymal stem cell (MSC)-derived exosomes and cell transplantation approaches. Among these, MSC-derived exosomes have demonstrated anti-calcific efficacy by inhibiting vascular and valvular cell osteogenic differentiation, suppressing calcium deposition, and modulating MAPK and NF-κB signaling pathways [77]. Additionally, VECs have been identified as key regulators of calcification, as they protect the VICs. Tissue engineering approaches include generating patient-specific cell sources through hiPSC (human inducible pluripotent stem cells)-derived VIC/VEC differentiation, reconstructing valve tissue structures using 3D hydrogels and bio-printing [78,89]. 

Seeding decellularized valve scaffolds with stem or progenitor cells has been proposed as a regenerative strategy to restore functional heart valve tissue, enabling recellularization of the native extracellular matrix architecture [79]. Collectively, these platforms extend beyond calcification inhibition, offering potential pathways toward functional valve regeneration and replacement.

### 5.4. Drug Repurposing

Drug repurposing strategies enable the application of existing drugs to new indications, thereby reducing development costs and timelines. In CAVD, antidiabetic agents and anti-inflammatory drugs have emerged as promising candidates.

Metformin, the most popular therapeutic drug in diabetes, consistently alleviated VICs calcification at the preclinical level by promoting autophagy-mediated degradation of Runx2 protein, thereby suppressing calcification [80]. Clinically, SGLT-2 inhibitors and GLP-1 receptor agonists are being investigated for their potential to slow aortic valve calcification. Notably, SGLT-2 inhibitors have been reported to reduce mortality and heart failure rehospitalization by 28% in post-TAVR patient cohorts [81,90]. 

Endothelial injury, immune cell infiltration, and cytokine secretion are key drivers of CAVD progression. Repurposing anti-inflammatory agents, such as IL-1β inhibitors and colchicine, has been explored [91]. In the LoDoCo2 trial, administration of low-dose colchicine (0.5 mg/day) to 5522 patients with chronic coronary artery disease for a median follow-up of 28.6 months reduced the incidence of MACE by 31% compared to placebo [72]. The mechanism of colchicine primarily involves suppression of the NLRP3 inflammasome through microtubule inhibition [92]. However, signals of increased non-cardiovascular mortality were observed, necessitating long-term safety evaluations [72].

Another approach, niclosamide, an FDA-approved anti-helminthic drug, demonstrated anti-calcification effects via mTOR inhibition in preclinical studies [82]. Although bisphosphonates showed efficacy in preclinical models, they failed to inhibit valve calcification in the SALTIRE II trial (NCT02132026) [5].

To date, robust evidence from randomized controlled trials specifically in CAVD patients remains insufficient. Drug repurposing thus represents a promising approach but requires further mechanistic elucidation and large-scale clinical validation.

### 5.5. Delivery Systems

Drug delivery strategies play a critical role in the success of novel therapeutics. Lipid nanoparticles (LNPs) and exosome-based platforms are particularly noteworthy. LNPs improve RNA stability and translation efficiency, while implantable drug delivery systems (IDDS) enable sustained drug release. Exosome-based carriers exhibit low immunogenicity and strong tissue tropism [83,84,93].

Targeted nanoparticles have been shown to selectively deliver drugs to calcified lesions, reducing calcification by 40% in animal models [84]. Magnetic nanoparticles targeting PAR2 receptors selectively delivered drugs to calcified aortic valves, effectively modulating inflammation and oxidative stress while suppressing calcification, highlighting the promise of active ligand–receptor binding nano-delivery systems for precision therapy in CAVD [85]. Non-viral nanocarriers are also anticipated to become pivotal in next-generation RNA therapeutics [83].

### 5.6. Future Directions

Future research is expected to expand toward multi-omics-based molecular target identification, tissue-engineered valve development, and patient-specific therapeutic strategies enabled by artificial intelligence (AI) and digital twin technologies [94]. Recent studies have reported increased calcium and phosphate levels accompanied by reduced magnesium and zinc concentrations in calcified valves. These mineral alterations indicate that dysregulation of mineral homeostasis may create a local metabolic environment that promotes valvular calcification. Such findings provide new insights into the pathophysiological mechanisms of valve calcification and suggest potential therapeutic strategies targeting mineral balance [95].

## 6. Challenges in the Development of Therapeutics for CAVD

### 6.1. Lack of Reliable Biomarkers

Despite the increasing prevalence of CAVD, no clinical biomarkers have been established to date, largely due to the absence of standardized diagnostic guidelines. Imaging-based parameters such as CT-derived AVC scores or Doppler echocardiographic indices are useful for assessing structural changes but fail to sensitively capture early pathological alterations or pharmacologic responses. Consequently, they remain insufficient as surrogate endpoints for therapeutic development [42]. Therefore, identifying molecular biomarkers that reflect early valvular remodeling and disease activity is crucial to improving diagnostic accuracy and enabling timely therapeutic intervention.

Recent multi-omics approaches—including proteomics, metabolomics, and transcriptomics—have been explored to identify novel biomarkers, yet none have reached clinical validation [7]. This absence of objective indicators hampers accurate assessment of therapeutic efficacy and disease prognosis, ultimately slowing the development of effective pharmacologic interventions.

### 6.2. Heterogeneous Pathogenic Mechanisms

CAVD is a multifactorial disease involving chronic inflammation, oxidative stress, fibrosis, and calcification. The predominance of specific mechanisms varies among patients, which explains why single-target therapeutic strategies often yield inconsistent outcomes. For instance, agents primarily aimed at inhibiting calcification may provide limited benefits in inflammation-dominant phenotypes [8]. Furthermore, genetic variations and differences in the tissue microenvironment contribute to heterogeneous drug responses, even for identical molecular targets [42]. This diversity underscores the necessity of personalized, mechanism-based treatment strategies that align with the patient’s specific pathophysiological subtype.

### 6.3. Structural Limitations in Clinical Trial Design

Clinical trials for CAVD face inherent challenges due to the disease’s slow progression and the lack of standardized, sensitive endpoints. Long-term follow-up is required to demonstrate therapeutic efficacy, but most study populations consist of elderly patients with multiple comorbidities, resulting in high attrition rates and slow recruitment [42].

Current surrogate measures, such as CT calcium scoring, reflect anatomical progression but correlate only weakly with clinical outcomes such as symptom improvement or survival [5]. To address these limitations, the field requires standardized quantitative metrics and integration of AI-based patient stratification systems to improve the interpretability and efficiency of CAVD trials [96].

## 7. Conclusions

CAVD has emerged as an actively regulated pathological process rather than a passive degenerative condition, involving chronic inflammation, fibrosis, and osteogenic differentiation of VICs [2,4,7]. However, clinical translation faces significant hurdles: the disease’s prolonged asymptomatic phase evades early detection, late referral due to diagnostic barriers remains prevalent, and clinical trials suffer from slow progression rates and inadequate surrogate endpoints that poorly correlate with clinical outcomes. Despite substantial advances in understanding its molecular mechanisms, no effective pharmacologic therapy is currently available. Surgical and transcatheter valve replacement remain the only treatment options, addressing hemodynamic consequences but not the underlying disease biology [29]. This unmet need highlights the importance of developing mechanism-based strategies that target the drivers of calcification and valvular remodeling.

Recent progress in molecular profiling, multi-omics technologies, and preclinical modeling has identified several candidate pathways, including IL-6, BMP2, Notch/Wnt, TNAP, and DPP-4 signaling, as potential therapeutic targets. However, clinical translation has been hindered by biological heterogeneity, lack of standardized endpoints, and, critically, the absence of reliable biomarkers capable of detecting early disease activity or pharmacologic response. This limitation prevents accurate assessment of therapeutic efficacy and impedes the development of disease-modifying agents.

Progress in CAVD drug development will depend on three parallel efforts: (1) discovery of early and quantitative biomarkers, (2) validation of multi-target therapeutic strategies, and (3) standardization of trial design and endpoint definitions. Emerging technologies such as AI and digital twin modeling, which enable virtual patient simulations to predict disease progression and drug responses, offer promising opportunities to optimize study design, enhance precision, and reduce trial duration [97]. Integrating these computational approaches into clinical pipelines could accelerate translation from molecular discovery to therapeutic application, ultimately transforming CAVD management from surgical intervention to precision-guided pharmacologic therapy.

Taken together, bridging molecular insights with clinical methodologies will be essential to achieve the first generation of effective, disease-modifying treatments for CAVD. Collaborative and multidisciplinary research efforts will play a pivotal role in moving the field toward this therapeutic milestone.

## Figures and Tables

**Table 1:** Summary of Clinical trials in CAVD.

No.	Trial/Sponsor	Indication	Study Phase/ Study Status	Patients No.	Primary End Points	Sponsor
1	AVADEC (NCT03243890)	Aortic sclerosis or mild-to-moderate calcific aortic valve stenosis	Phase unspecified Completed	304	Change in CT-based aortic valve calcification after 2 years	Odense Univ Hospital
2	EAVaLL (NCT02109614)	Aortic sclerosis or mild calcific aortic stenosis associated with elevated Lp(a)	Early Phase 1 Withdrawn	0	Change in aortic valve calcification at 2 years	McGill University
3	PCSK9-AS (NCT03051360)	Mild–moderate calcific aortic valve stenosis	Phase 2 Unknown	140 (Estimated)	Change in AS progression (Vmax, AVA, and AVC) over 2 years	SNU Hospital
4	SALTIRE II (NCT02132026)	Mild-to-moderate calcific aortic valve stenosis (tricuspid or bicuspid)	Phase 2 Completed	150	Change in CT-based aortic valve calcium at 2 years	University of Edinburgh
5	BASIK2 (NCT02917525)	Bicuspid aortic valve with early calcific aortic valve disease	Phase 2 Completed	44	Change in valvular microcalcification activity measured by ^18^F-NaF PET/MR after 6 months	Maastricht UMC+
6	DIP-CAVD (NCT04055883)	Mild–moderate calcific aortic valve stenosis	Phase 2 Completed	228	Change in AVC volume (CT) from baseline to 96 weeks	Dong-A ST Co., Ltd.
7	LoDoCo2 (ACTRN12614000093684)	Coronary heart disease diagnosed by coronary angiography or CT coronary angiogram	Phase 3 Completed	5522	Change in HR and 95% CI (derived from a Cox model adjusted for treatment group) at 12 months	National Health and Medical Research Council of Australia
8	COPAS-Pilot (NCT05253794)	Mild–moderate calcific aortic valve stenosis	Phase 2 Recruiting	24 (Estimated)	Change in ^18^F-NaF PET–measured valvular microcalcification activity over 6 months	Ottawa Heart Institute Research Corporation
9	NCT05875675	Mild-to-moderate calcific aortic valve stenosis	Phase 2 Not yet recruiting	100 (Estimated)	Change in aortic stenosis severity (peak transaortic velocity) over 104 weeks	Wuhan Union Hospital
10	Lp(a) FRONTIERS CAVS (NCT05646381)	Mild–moderate calcific aortic valve stenosis with elevated Lp(a)	Phase 2 Recruiting	502 (Estimated)	Change in peak aortic jet velocity and CT-measured aortic valve calcium score over 3 years	Novartis Pharmaceuticals
11	CaLIPSO (NCT02966028)	Mild–moderate calcific aortic valve stenosis	Phase 2b Completed	274	Change in CT-measured coronary artery calcium and aortic valve calcification over 52 weeks	Sanifit Therapeutics S.A.
12	EVOID-AS (NCT05143177)	Mild–moderate calcific aortic valve stenosis	Phase 2/3 Recruiting	580 (Estimated)	Change in CT-based AVC at 104 weeks	REDNVIA Co., Ltd.
13	ASTRONOMER (NCT00800800)	Mild–moderate asymptomatic aortic stenosis	Phase 3 Completed	269	Annualized change in peak aortic jet velocity (Vmax) to assess aortic stenosis progression	AstraZeneca
14	SEAS (NCT00092677)	Mild–moderate asymptomatic aortic stenosis	Phase 3 Completed	1873	Time to first major cardiovascular event (composite of CV death, AVR, MI, or stroke).	Organon and Co.
15	CHIANTI (NCT05162742)	Mild–moderate calcific aortic valve stenosis	Phase 3 Active, not recruiting	150 (Estimated)	Change in CT-AVC and Vmax/AVA at 24 months	Radboud University Medical Center
16	EPISODE (NCT04968509)	Mild–moderate calcific aortic valve stenosis	Phase 3 Recruiting	160 (Estimated)	Annualized change in peak aortic jet velocity (Vmax) over 2 years	Beijing Anzhen Hospital
17	KATALYST-AV (NCT07001800)	Moderate calcific aortic valve stenosis	Phase 3 Recruiting	1410 (Estimated)	Change in CT-AVC at 6 months and change in peak VO_2_ at 12 months	Kardigan, Inc.
18	ARBAS (NCT04913870)	Mild–moderate calcific aortic valve stenosis	Phase 4 Recruiting	144 (Estimated)	Change in peak aortic jet velocity (Vmax) and left ventricular remodeling over 2 years	IUCPQ-University Laval

**Table 2:** Investigational therapeutic approaches in CAVD.

Modality	Example Agents/Strategies	Development Stage	Key Findings	Limitations	Ref.
Small molecules	SNF 472	Preclinical Phase 2b	Inhibits hydroxyapatite crystallization	Effect on valvular calcification unproven	[64]
Gene therapy	ENPP1 mutation	Preclinical	Linked to ectopic calcification activity ↓	Mouse–human mismatch	[73]
	miRNA modulators (miR-34a, miR-204, lncRNA H19)	Preclinical	Prevented VIC calcification	Preclinical only	[74]
	IncRNA H19 inhibition	Preclinical	Suppresses osteogenic differentiation via NOTCH1 restoration	Preclinical only	[75]
	CRISPR-Cas9 gene editing	Preclinical	Direct correction of PCSK9 and CVD genes	Off-target, ethical & delivery issues	[76]
Cell therapy	MSC-derived exosomes	Preclinical	Inhibited VSMC calcification via MAPK/NF-κB modulation	Tested only in vascular cells	[77]
	hiPSC-derived VIC/VEC differentiation	Preclinical	Generated patient-specific valve cell sources	Needs functional validation in vivo	[78]
	3D hydrogel-based VIC/VEC cultures	Preclinical	Recreates native valve microenvironment and supports calcification modeling	Limited tissue complexity and long-term stability	[79]
Drug repurposing	DPP-4 inhibitor	Phase 2/3	Regulator of VICs osteogenic differentiation	A small cohort of patients	[34,35,68]
	Metformin	Preclinical	↓ Calcification, ↓ Runx2, ↑ autophagic flux (Atg3/7, LC3-II)	In vitro only; need in vivo/clinical validation	[80]
	SGLT2 inhibitors	Observational	↓ Mortality and HF post-TAVR	Observational data only	[81]
	Colchicine	Phase 3	↓ CV events 31%	Possible ↑ non-CV mortality	[72]
	Niclosamide	Preclinical	mTOR inhibition, anti-calcific effect	Only animal /in vitro studies	[82]
	Bisphosphonates	Phase 2	No efficacy in slowing CAVD progression	Negative trial result	[5]
Delivery systems	Lipid nanoparticles (LNPs)	Early clinical	Improved RNA delivery	Early data only	[83]
	Implantable drug delivery systems (IDDS)	Preclinical	Controlled release demonstrated	No clinical evidence	[83]
	Exosome carriers	Preclinical	Low immunogenicity, tissue-friendly	Translational stage unclear	[84]
	PAR2-targeting magnetic nano-cargoes	Preclinical	Inhibited VIC osteogenic differentiation, reduced calcification	Strong hemodynamic environment challenge, safety needs validation	[85]

↓: decreased, ↑: increased (indicates the direction of change in activity, expression, or calcification).

## Data Availability

No new datasets were generated in this study. Data supporting the analyses were obtained from publicly available sources, including ClinicalTrials.gov (accessed on 2 September 2025) and published literature indexed in PubMed. Additional trial information was verified using sponsors’ websites or official releases.
